# Advanced machine learning techniques reveal multidimensional EEG abnormalities in children with ADHD: a framework for automatic diagnosis

**DOI:** 10.3389/fpsyt.2025.1475936

**Published:** 2025-02-14

**Authors:** Ying Mao, Xuchen Qi, Lingyan He, Shan Wang, Zhaowei Wang, Fang Wang

**Affiliations:** ^1^ Department of Special Examination, Shaoxing Peoples’ Hospital, Shaoxing, Zhejiang, China; ^2^ School of Medicine, Shaoxing University, Shaoxing, Zhejiang, China; ^3^ Department of Neurosurgery, Shaoxing People’s Hospital, Shaoxing, Zhejiang, China; ^4^ Department of Neurosurgery, Sir Run Run Shaw Hospital, Zhejiang University School of Medicine, Hangzhou, Zhejiang, China; ^5^ Department of Traditional Chinese Medicine, Shaoxing People’s Hospital, Shaoxing, Zhejiang, China; ^6^ Department of Neurology, Shaoxing People’s Hospital, Shaoxing, Zhejiang, China

**Keywords:** attention deficit hyperactivity disorder (ADHD), electroencephalography (EEG), functional connectivity (FC), feature selection, machine learning

## Abstract

**Introduction:**

Attention deficit hyperactivity disorder (ADHD) is a prevalent neurodevelopmental disorder that affects attention, impulse control, and multitasking abilities in children and adults. Understanding electroencephalography (EEG) characteristics of children with ADHD can provide new diagnostic tools and personalized treatment plans. This study aims to explore potentially promising EEG features using advanced machine learning techniques and feature selection technique (i.e., SHapley Additive exPlanations (SHAP) algorithm) to reveal brain function abnormalities between pediatric children with ADHD and healthy controls (HC) in a data-driven manner.

**Methods:**

Multidimensional EEG characteristics were extracted from multiple domain (including power spectral density (PSD), fuzzy entropy (FuzEn), and functional connectivity features of mutual information (MI)) using a publicly-available dataset. Then, four widely-employed machine learning algorithms (including random forest (RF), XGBoost, CatBoost, and LightGBM) were used for classification calculations, and the SHAP algorithm was then used to assess the importance of the contributing features to interpret the model’s decision process.

**Results:**

The results showed that the highest classification accuracy of 99.58% for pediatric ADHD detection was obtained with the CatBoost model based on the optimal feature subset of 206 features (PSD/FuzEn/MI = 53/5/148). According to the optimal feature subset statistics, there is an increase in the power of theta, alpha, and beta rhythms, an elevated power ratio between theta and beta (theta/beta ratio, TBR), and reorganization of whole-brain functional connectivity across all frequency bands in children with ADHD, primarily characterized by enhanced functional connectivity.

**Discussion:**

We showed that EEG features was effectively extracted by machine learning methods, which could play a critical role in classification between pediatric ADHD and HC. These findings provide strong evidence for revealing the electrophysiological mechanisms through multidimensional EEG characteristics and move a step forward towards future automatic diagnosis of ADHD.

## 1 Introduction

Attention Deficit Hyperactivity Disorder (ADHD) is a prevalent neurodevelopmental disorder characterized by inattention, hyperactivity, and impulsivity. It typically manifests in childhood and often persists into adulthood, affecting learning, social relationships, and daily functioning (1, 2). According to (3–5), ADHD affects about 5 – 12% of children and adolescents globally despite different diagnostic criteria and reporting practices across regions (6). The severity of ADHD symptoms can differ greatly. In severe cases, individuals might face academic struggles due to attention deficits and hyperactive behavior. Socially, subjects with ADHD present challenges as impulsivity and inattentiveness that may lead to challenge to form as well as maintain friendships, often resulting in social rejection (7). Additionally, ADHD is often associated with other emotional and behavioral issues such as anxiety, oppositional defiant disorder, and conduct disorder (8). These co-occurring conditions can aggravate daily challenges, including poor job performance, marital problems, and a higher risk of traffic accidents due to impulsivity and inattention (9, 10). Research indicates that individuals with ADHD are at a greater risk of substance abuse and addiction, which increases health risks. Take into account of these undesirable consequences, early identification of ADHD could promote early intervention which may significantly reduce the negative impacts of ADHD, improve academic performance, social interactions, and ultimately overall quality of life.

According to the Diagnostic and Statistical Manual of Mental Disorders, 5th Edition (DSM-5) (11), the diagnosis of ADHD should be based on observing behavior related to attention, activity level, and impulsivity. However, behavioral assessment is subjective and may not fully capture the complexity of ADHD. Parents, teachers, and others known to the child are usually involved in the treatment plan to provide additional information that may contribute to accurate diagnosis. The symptoms of the child are checked against standardized behavioral scales, but the results again rely on the subjective evaluation of the examiner, and variations cannot be completely eliminated. Therefore, there is a need to develop objective markers for accurate and reliable diagnosis of ADHD. In this regard, the widely-used subjective questionnaires may not appreciate parts of the real condition of the patient being assessed. Moreover, questionnaires may not be applied universally among persons of different cultures and languages, and this affects diagnosis or its uniformity (12). To this end, behavioral assessment and neuroimaging are commonly used to provide additional information (13). Nevertheless, behavioral assessment relies on subjective reports that may not fully capture the disorder’s complexity, whereas neuroimaging (such as functional magnetic resonance imaging (fMRI), positron emission tomography (PET), and near-infrared spectroscopy (NIRS)) are typically costly and require specialized expertise. An objective and reliable analysis framework that could provide reliable and accurate diagnosis of ADHD through incorporating cost-effective neuroimaging technique and advanced machine learning methods is therefore of great importance.

Machine learning has apparent potentials for the diagnosis of mental disorders (14). Indeed, early diagnosis of mental disorders could be done by analyzing and integrating large amounts of clinical characteristics, biomarkers, and neuroimaging data through machine learning. In fact, data from neuroimaging reveal abnormal patterns and features that may possibly explain the underlying etiology of the disorder. In comparison with the hypothesis-driven clinical studies, machine learning is good at processing and analyzing high-dimensional multidimensional data to discover possible patterns and complex relationships of mental disorders (15–17). Such analyses give insights into the deeper pathophysiological mechanisms of diseases from a data-driven fashion, which may therefore provide a scientific rationale for developing novel diagnostic methods. As such, the application of machine learning has several benefits for ADHD detection. It improves diagnostic accuracy by providing subtle differences in brain structure and function that otherwise would be difficult to obtain using conventional means. Further, machine learning contributes to the overall understanding of ADHD by elaborating differences in functional connectivity, frequency characteristics, and other brain dynamics in the brain. This deeper understanding helps in creating more focused and effective diagnosis methods of ADHD.

Heuristically, EEG offers millisecond temporal resolution, enabling real-time acquisition of fast neural processes crucial for studying dynamic brain activity (18). Its non-invasive nature, portability, and relative affordability have led to its widespread use in clinical and laboratory settings. Of note, EEG has already been far-reaching in research and diagnostics of ADHD (19, 20). For instance, children with ADHD typically exhibit higher theta and lower beta powers compared to healthy controls (HC) (21, 22). The power ratio between theta and beta waves (theta/beta ratio, TBR) is often higher in children with ADHD than those of normal controls, making TBR a potential biomarker for ADHD. In addition to power spectral density analysis, nonlinear dynamics of EEG signal offers new insights. Fuzzy entropy (FuzEn) is a nonlinear metric of the complexity and uncertainty of a time series (23, 24). By measuring the EEG FuzEn, researchers can quantify the state of nervous system functioning, making it a valuable supplementary diagnostic tool (25). This ability to assess complexity is particularly useful in estimating the complexity features of ADHD EEG signals. Functional connectivity analysis, using mutual information (MI), is another key approach. MI is a non-parametric statistical method that measures the shared information between two random variables, uncovering complex nonlinear relationships (26, 27). In ADHD researches, characteristic abnormalities in MI patterns across various EEG rhythms can define the functional connectivity features that may potentially facilitate clinical diagnosis. Analyzing functional connectivity features provides more information about the neurophysiological mechanisms of ADHD. In the context of ADHD, both resting-state and task-state functional connectivity offer valuable insights. Resting-state functional connectivity measures the correlation of activity time series while subjects are at rest (28), revealing intrinsic connectivity networks that are more permanent and correlate well with anatomical connectivity. In contrast, task-state functional connectivity assesses the interactions between brain regions during specific cognitive tasks (29). For children with ADHD, task-state connectivity often shows hyper-connectivity in certain frequency bands (30), which may reflect exaggerated interactions between brain regions during cognitive processing. This hyper-connectivity can be more effective for ADHD diagnosis. In summary, such findings from EEG data analysis enable clinicians to better understand the neurophysiological underpinnings of ADHD, allowing for more effective treatment strategies tailored to individual patient needs, ultimately enhancing outcomes in terms of attention and behavioral control (31).

In this work, a machine learning analysis framework is developed to extract salient EEG features for accurate diagnosis of ADHD, which may lead to a deep understanding of important abnormal neural mechanisms in children with ADHD. The employed features include PSD, FuzEn, and MI, which are fed into machine learning classifiers to obtain the highest classification accuracy. Feature selection was done using SHapley Additive exPlanations (SHAP) feature selection. Signal analysis for the understanding and interpretation of physiological significance of ADHD was then conducted based on the selected contributing features. The suggested analysis framework will help to make explicit the important neurobiological characteristics of ADHD and may move a step forward towards future automatic diagnosis of ADHD.

## 2 Materials and methods

### 2.1 Dataset introduction

A publicly-available dataset was utilized in this study (32). The dataset includes two groups of children. One group consists of 61 children diagnosed with ADHD (male/female = 48/13, age = 9.62 ± 1.75 years old), strictly diagnosed by psychiatrists. The healthy group consists of 60 gender- and age-matched HC children (male/female = 50/10, age = 9.85 ± 1.77 years old). No children in the HC group had any mental disorders, a history of epilepsy, or reported high-risk behaviors. All participants were school-aged and right-handed. The data collection was approved by the Institutional Review Board and Ethical Committee of Tehran University of Medical Sciences. Written informed consent was obtained from each participant and/or their parents. Further details pertaining to the dataset could be referred to the original work (33).

Given the significant deficits in visual attention among children with ADHD, a visual attention task was designed and EEG signals were collected during the task. Briefly, the task involved showing children a series of cartoon character images and requiring them to accurately count the number of characters in each image. The images were designed to be large enough for all children to easily identify and count. To ensure the continuity of stimulation and the effectiveness of the task, each image seamlessly transitioned to the next immediately after the response. This formed a smooth cognitive visual task flow. Therefore, the duration of the entire EEG recording was not fixed but flexibly adapted to the response speed of each child. This setup more accurately captured the dynamics of their neural activity during the visual attention task. EEG data were collected from 19 channels (including Fp1, Fp2, F3, F4, F7, F8, Fz, C3, C4, Cz, P3, P4, Pz, T3, T4, T5, T6, O1 and O2) according to the standardized international 10-20 system with a sampling rate of 128 Hz. A1 and A2 electrodes were used as earlobe reference points. During the recordings, the electrode impedances were kept below 5 kΩ.

### 2.2 Feature extraction

**Figure 1:**
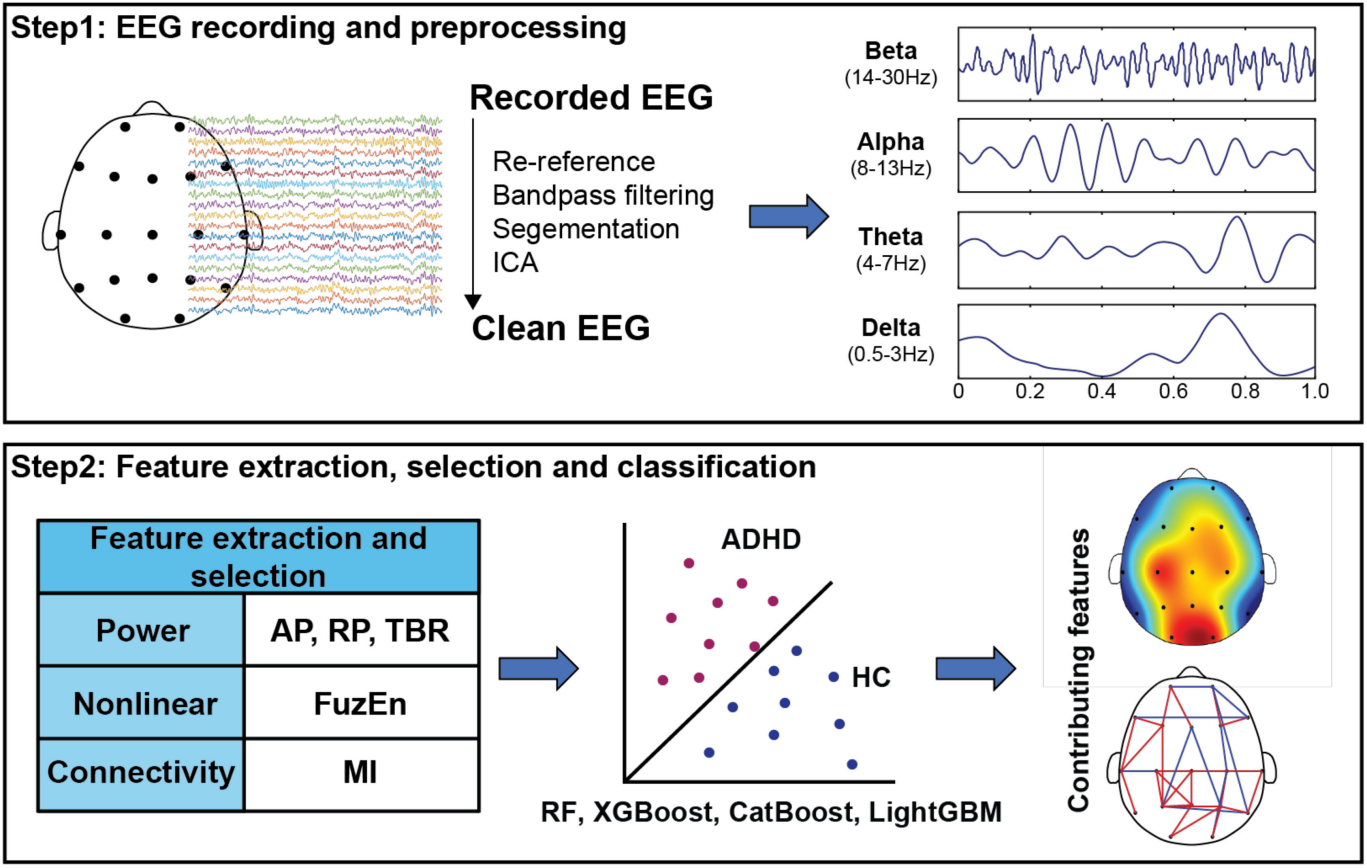
The analysis flow chart of this study.

In **Figure 1**, we showed the analysis flowchart of the current work. Specifically, the 19-channel EEG signals were segmented into 4-second segments. A 4^th^ order Butterworth filter was applied to band-pass filter each segment within the 0.5 – 30 Hz range to remove noise from low-frequency respiratory waves and high-frequency electromyography waves. Independent component analysis (ICA) was then used to identify and remove the noise components. For the subsequent calculation, EEG data were filtered into four conventional canonical frequency bands (including delta: 0.5 – 3 Hz, theta: 4 – 7 Hz, alpha: 8 – 13 Hz, beta: 14 – 30 Hz).

From these extracted rhythm bands, a total of 931 features were derived: PSD (absolute power (AP), relative power (RP), TBR), nonlinear dynamics (FuzEn), and functional connectivity (MI). The number of features for RP, AP, and FuzEn were each 76 (19 channels × 4 frequency bands), the number of features for the theta/beta power ratio was 19, and the number of functional connectivity features was 684 (19 channels × (19 channels – 1) × 4 frequency bands/2). Detailed formulas of PSD features and FuzEn features can be found in the literature (34). MI was estimated between each pair of EEG channels:


(1) $$\text{MI}(X,Y)=\sum_{\text{X},\text{Y}}P(X,Y)\log\frac{P(X,Y)}{P(X)P(Y)}$$

where *X* and *Y* represent the EEG signals from two channels, P(X) denotes the probability that $X=X_{i}$ occurs, P(Y) denotes the probability that $Y=Y_{i}$ occurs, and P(X,Y) denotes the probability that ( $X=X_{i},\;Y=Y_{i}$ ) occur at the same time, i.e., the joint probability. By using a standardized 10-20 electrode placement system, preprocessing the EEG signal, calculating functional connectivity using MI, and applying the SHAP algorithm for feature selection and model evaluation, this study mitigates the impact of volume conductor effects on EEG functional connectivity calculations to some extent.

### 2.3 Machine learning models

In the current work, 10-fold cross-validation was adopted. Specifically, the dataset was randomly divided into 10 segments where 9 segments were used as training sets, while the remaining 1 segment served as the test set. Four widely-used ensemble learning models (i.e., random forest (RF), XGBoost, CatBoost, and LightGBM) were employed for ADHD classification.

1. RF: RF is an ensemble learning approach designed to enhance the accuracy and robustness of prediction by building multiple decision trees and afterward integrating their outcome predictions to make the final decisions. RF trains every decision tree independently based upon a randomly selected subset of the dataset, known as bootstrap sampling. Moreover, at each split node, it selects a random set of features in order to choose the best split. These dual randomness help in reducing overfitting by making sure that the individual trees are diverse and uncorrelated. RF finally makes a prediction by aggregating the predictions from all the trees, often using majority voting for classification tasks. RF has many advantages, such as easy implementation, fast training, and resistance to outliers and noisy data. It is suitable for high-dimensional data because a subset of features can be randomly selected for splitting, which helps to reduce the correlation between individual trees and improves the generalization of the model. For the above reasons or advantages, it has wide applications in data science and machine learning.
2. XGBoost: XGBoost is an integrated learning algorithm based on the Gradient Boosting framework, which builds powerful predictive models by combining multiple weak learners (usually decision trees).The basic principles of XGBoost include: using gradient boosting to minimize the loss function by incrementally adding new models (trees) to minimize the loss function, and adjusting the new model according to the error of the previous model in each step to improve the prediction performance; at the same time, XGBoost introduces L1 and L2 regularization terms to prevent overfitting, and accelerates the training process through parallel computation, which performs especially well on large datasets.
3. CatBoost: CatBoost mitigates overfitting during training through Symmetric Trees and Ordered Boosting, which are the components enhancing model generalization and accuracy. One of the top characteristics is that it natively handles categorical features and missing values, hence much reducing the amount of preprocessing needed at the beginning of a project. For real-world datasets, CatBoost often has such complexities, providing robust performance and ease of use for the practitioner. Its ability to automatically process missing values and categorical features reduces the workload of data preprocessing, while its efficient feature processing and prediction speed enables faster and more accurate extraction of diagnostic-value features in EEG signal classification tasks, which provides a strong support for the realization and development of EEG signal technology.
4. LightGBM: LightGBM is a fast and efficient gradient boosting framework that enables rapid training and accurate predictions on large-scale datasets. The histogram-based learning algorithm is applied here along with a number of optimization techniques like gradient-based one-side sampling and parallel leaf splitting. LightGBM is particularly efficient when dealing with high-dimensional data.

### 2.4 Evaluation metrics

In order to quantitatively assess the performance of the classification models, the following evaluation metrics were adopted in this study:

1. Accuracy is defined as the proportion of instances correctly predicted (both positive and negative) compared to the total number of instances.


(2) $$ACC=\frac{TP+TN}{TP+FP+TN+FN}$$

2. Precision is the ratio of true positive samples to the total number of samples predicted as positive by the model.


(3) $$PR=\frac{TP}{TP+FP}$$

3. Recall is defined as the proportion of true positive samples that the model successfully identifies as positive among all actual positive samples.


(4) $$RE=\frac{TP}{TP+FN}$$

4. The F1 score, which is the harmonic mean of precision and recall, is used to assess the accuracy of a binary classification model.


(5) $$F1=\frac{2PR\times RE}{PR+RE}$$

5. AUC (Area Under the ROC Curve) refers to the area under the Receiver Operating Characteristic (ROC) curve, which represents the model’s ability to correctly classify positive samples with a higher probability than negative samples. The value of AUC ranges from 0 to 1, with higher values indicating superior model performance.

In Equations 2-5, TP (True Positive) indicates the count of positive instances correctly predicted as positive by the model. FP (False Positive) denotes the count of negative instances incorrectly predicted as positive. TN (True Negative) signifies the number of negative instances accurately identified as negative by the model, indicating correct identification of actual negative instances. FN (False Negative) represents the count of positive instances incorrectly predicted as negative by the model.

### 2.5 Feature selection algorithms

Due to the inclusion of a total of 931 features, there was a potential risk of feature redundancy. Consequently, this study performed feature selection based on all features. Feature selection method was chosen due to its ability to reduce data dimensions, extract the most representative features, and improve model performance and generalization from high-dimensional data. SHAP is a high-level model interpretation method, based on the theory of game values, for explanation of the prediction results from any machine learning model (33). It provides an intuitive way to interpret how the models make decisions and allows deep attribution analysis in order to discover intricate mechanisms of model predictions. SHAP gives a global perspective that is important to develop an overall understanding of how a model is behaving, particularly in the case of identifying which features are most important to its predictions. Its global interpretation therefore ranks input features by their importance for feature selection, model optimization, and further scientific investigation. With this ranking, we can determine which features contribute the most to the model’s prediction and, therefore, focus on these key features for further analysis or consider higher weights for them while making the model. Conversely, features contributing minimally or negligibly can be removed during training so as to ensure that it becomes more efficient and interpretable. Because of the global explanation of SHAP in this study, it will be able to determine which EEG features are most strongly correlated with ADHD predictions. That way, it will help researchers and clinicians further understand the complex etiology of the disorder. Of note, we opted the data-driven feature selection over traditional statistical comparisons for the following reasons: 1) feature selection serves as an effective approach for handling high-dimensional data whereas multiple independent statistical comparisons would inevitably lead to the well-known multiple comparison problems; 2) the SHAP algorithm could provide interpretable explanations about the decision-making process of the classification models that would facilitate the revealing of the underlying neural mechanisms.

## 3 Results

### 3.1 Classification performance and feature selection

We first assess the classification performance. The results for the four ensemble learning models about the classification task were depicted in **Table 1**. In general, satisfactory classification performance was achieved for four models. Further comparison showed a superior overall performance of CatBoost model (ACC = 99.58 ± 0.29%, RP = 99.67 ± 0.46%, RE = 99.44 ± 0.79%, F1 = 99.55 ± 0.34%, and AUC = 99.44 ± 0.39), followed by Light GBM, XGBoost and RF.

**Table 1:** Model Performances for ADHD classification.

Models	ACC (%)	PR (%)	RE (%)	F1 (%)	AUC
CatBoost	**99.58 ± 0.29**	**99.67 ± 0.46**	**99.44 ± 0.79**	**99.55 ± 0.34**	**99.44 ± 0.39**
LightGBM	99.52 ± 0.20	99.61 ± 0.43	99.37 ± 0.75	99.49 ± 0.26	99.37 ± 0.29
XGBoost	99.22 ± 0.32	99.37 ± 0.65	98.97 ± 1.14	99.16 ± 0.42	98.97 ± 0.42
RF	97.64 ± 0.36	98.17 ± 1.78	96.84 ± 3.17	97.44 ± 0.82	96.84 ± 0.45

Bold value indicates best performance.

Given that the superior classification performance was obtained using the CatBoost model, the feature selection via SHAP algorithm was conducted on the CatBoost model. Specifically, the SHAP values for all features were obtained and ranked in descending order of importance. Subsequently, features were iteratively added as inputs to retrain the CatBoost model based on their importance. In each iteration, one feature was added according to its ranked importance until all features were included, resulting in a series of accuracy evaluations. The best classification performance was achieved when the number of input features of the CatBoost model reached 206. In **Table 2**, we showed the distribution of the contributing features according to their types. Specifically, most of the contributing features were functional connectivity features (148 out of 206), where theta (N = 40), alpha (N = 34) and beta (N = 57) frequency bands exhibited predilection. As for the power spectral and Fuzzy features, we found 29 AP features, 14 RP features, 10 TBR features and 5 FuzEn features mainly resided in high frequency bands.

**Table 2:** Distribution of features in the optimal feature subset.

Feature Types	Delta	Theta	Alpha	Beta
AP	0	7	8	14
RP	0	0	1	13
TBR	10			
FuzEn	0	0	2	3
MI	17	40	34	57

### 3.2 Spatial and spectral characteristics of the contributing features

**Figure 2:**
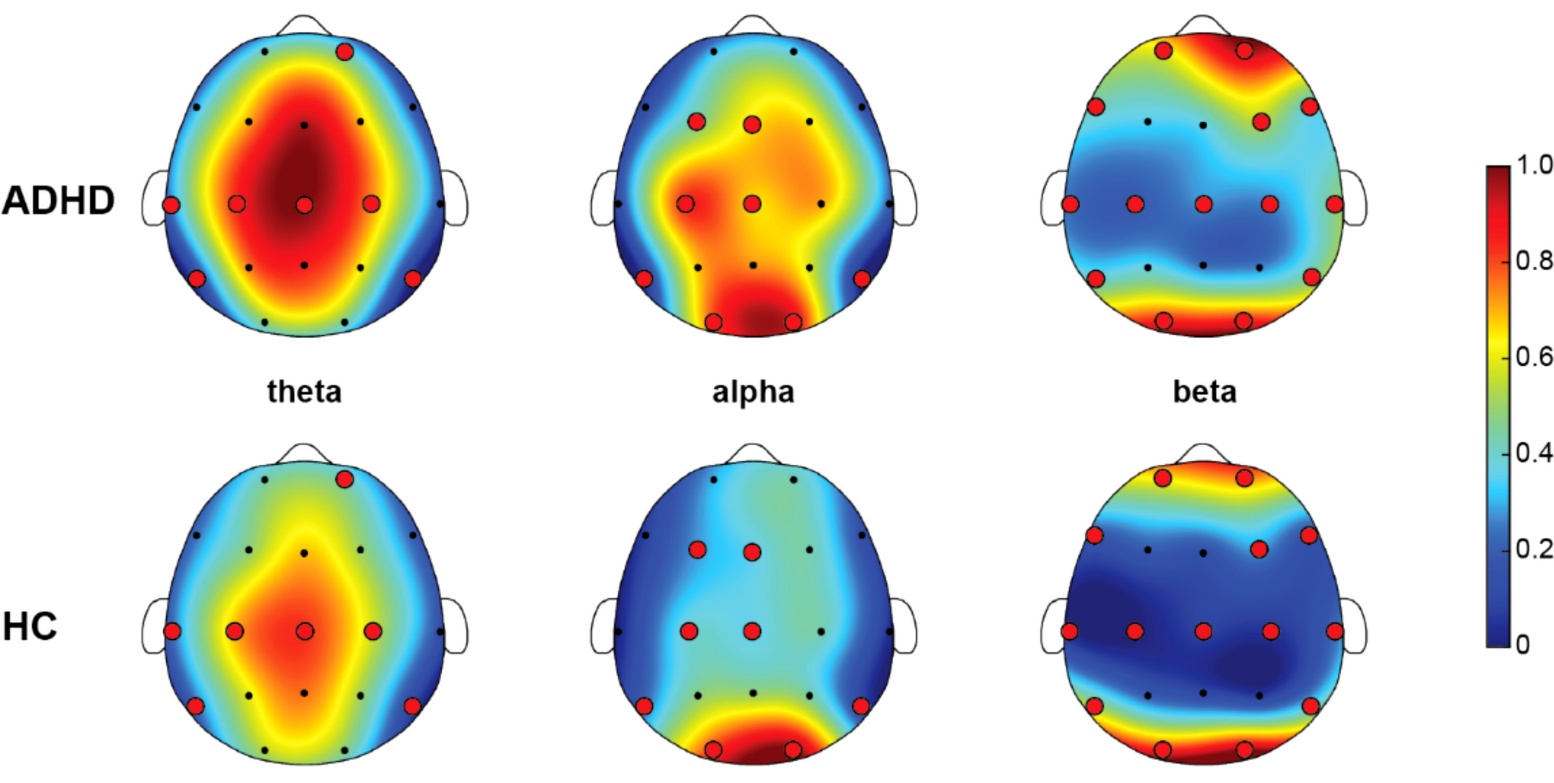
Topographic map of absolute power (AP) for theta, alpha, beta rhythms between ADHD and HC. Specifically, the red areas represent high power, and the blue areas represent low power. The red dots refer to the selected EEG channels based on SHAP method. All EEG channels of each rhythm were normalized to between 0 and 1 among ADHD and HC, resulting in the color bar that was between 0 and 1.

**Figure 2** illustrated the distribution of averaged AP within the optimal feature subset. The figure showed the AP distribution across three frequency bands (theta, alpha, beta) for children with ADHD and HC groups. In the theta band, the EEGs of the ADHD group exhibited high power (red areas), particularly in the frontal regions. HC group also displayed relatively higher theta band power in the frontal regions but at a lower overall power level (shades of red-yellow). In the alpha band, the ADHD group showed higher power in the frontal and top regions, depicted by yellow and orange areas. HC display power concentrated in the frontal regions with lower overall power levels (shades of blue-green). In the beta band, the ADHD group presented higher power in the top regions, indicated by yellow areas. HC exhibited lower beta band power levels overall, shown by blue and green areas. These scalp maps indicated that brain activity in the theta and alpha bands is increased in the ADHD group compared to the HC group, while differences in beta band activity were smaller. Overall, children with ADHD depicted more active brain activity in the low-frequency bands, which may be related to their symptomatic characteristics.

**Figure 3:**
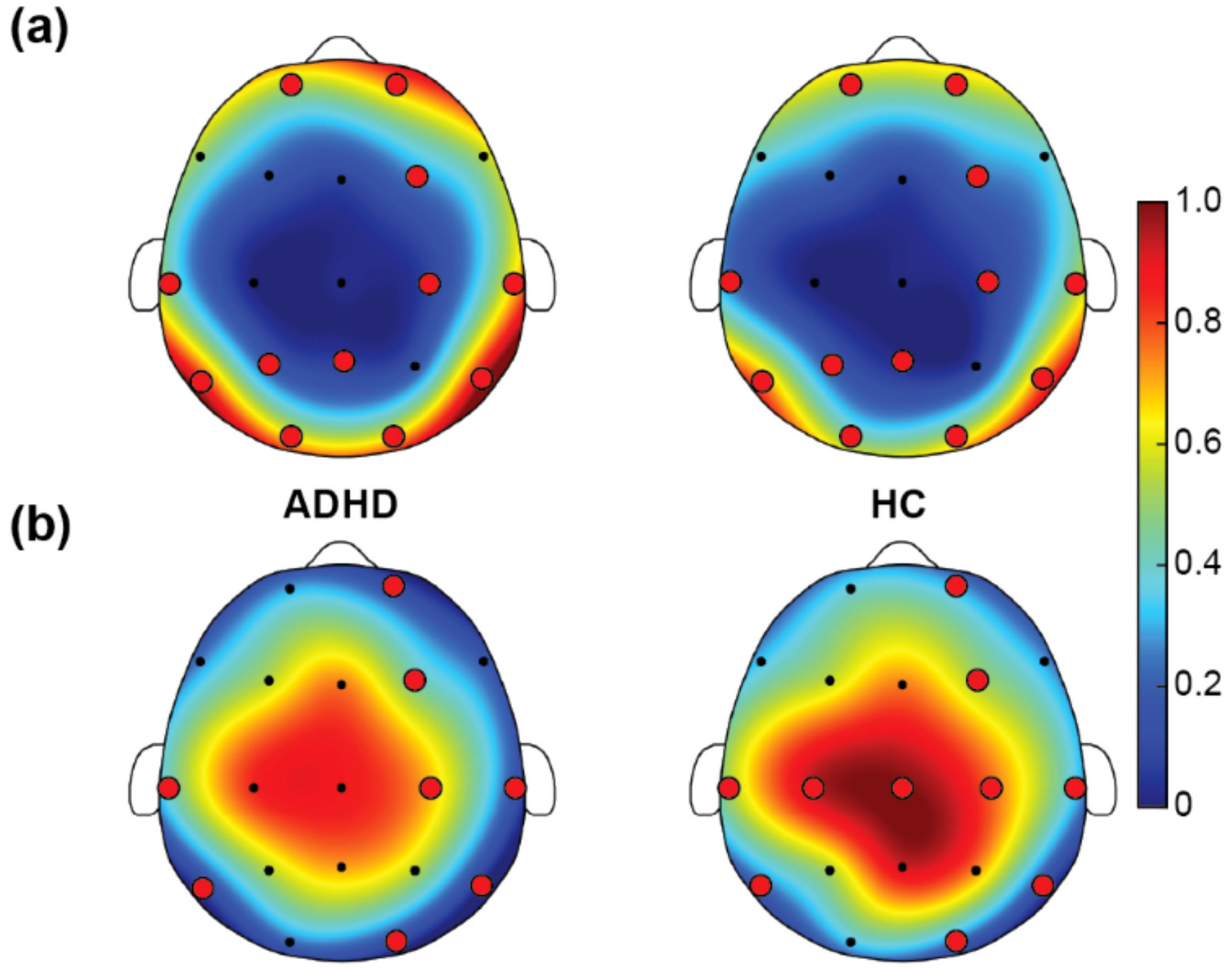
Results of power ratio. **(A)** Relative power (RP) of beta, and **(B)** theta/beta power ratio (TBR). In the figure, the red and yellow regions indicate high RP and TBR, and the blue and green regions indicate low RP and TBR. The red dots refer to the selected EEG channels based on SHAP method. For the power ratio comparisons between ADHD and HC, both RP and TBR were 0-1 normalized across all EEG channels between the two groups, resulting in the color bar that was between 0 and 1.

**Figure 3A** presented the RP in the beta band for children with ADHD and HC. In the beta band, the ADHD group’s RP was mainly concentrated in the peripheral areas of the scalp, especially in the top and posterior brain regions, indicating a little higher beta power in these areas. **Figure 3B** displayed the TBR results. ADHD group showed higher TBR in the frontal regions, depicted by red areas. This indicated that in these regions, theta band power was much higher than beta band power. HC illustrated lower overall ratios on the same scale, mainly concentrated in the middle areas, appearing in yellow and orange areas, indicating a relatively balanced power ratio between theta and beta bands. In summary, the ADHD group showed a little higher RP in the beta band and higher TBR, especially in the frontal regions. These differences may reflect the neurophysiological characteristics of children with ADHD in different frequency bands, aiding in the further understanding of the neural mechanisms of ADHD.

**Figure 4:**
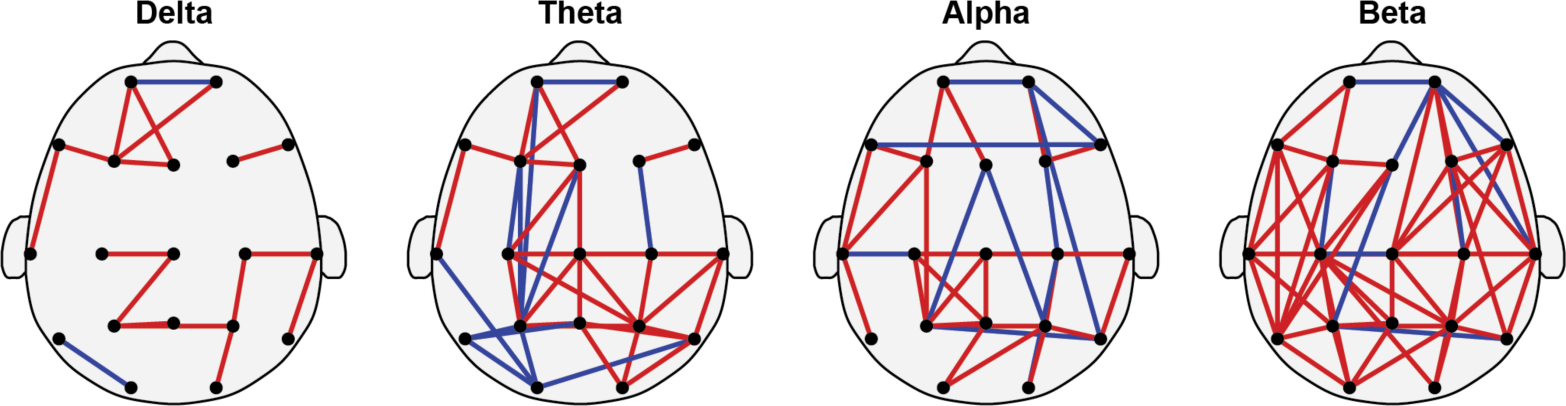
Brain functional networks of delta, theta, alpha, and beta rhythms. Specifically, the red edges indicate increased values of mutual information (MI) in the ADHD group, i.e., enhanced functional connectivity. And the blue edges indicate reduced values of mutual information in the ADHD group, i.e., weakened functional connectivity. (Note: The proportion of enhanced functional connectivity in ADHD is 15/17 = 88%, 28/40 = 70%, 24/34 = 71%, 48/57 = 84%).

**Figure 4** showed the differences in brain functional connectivity between children with ADHD and HC across delta, theta, alpha, and beta rhythms. Red edges indicate increased MI values in ADHD compared to HC, while blue edges signify reduced MI values. It is noteworthy mentioning that here the increased and reduced MI values were not determined via traditional statistical comparisons. Instead, these edges were identified as increased or reduced via comparing the mean value of those selected individual feature. In the delta band, ADHD shows increased MI in frontal and top regions, with minimal decrease compared to HC. Increased connectivity in ADHD, particularly in frontal and central regions, with some reduced connectivity areas were observed in the theta band. Alpha rhythms display evenly distributed increased MI in multiple ADHD regions. Beta rhythms show markedly enhanced connectivity in posterior brain regions of ADHD compared to HC. Overall, children with ADHD exhibit increased MI values in multiple brain regions, especially under theta and beta rhythms.

## 4 Discussion

### 4.1 Satisfactory classification performance

To date, multiple studies had shown the feasibility of utilizing EEG features in the diagnosis of ADHD. Different types of features could decode EEG signals from different perspectives, but not all features are relevant to diagnosis. This study obtained an optimal feature subset of 206 features, achieving a superior classification accuracy of 99.58 ± 0.29%. The classification performance was mostly superior in comparison with several recent studies [i.e., Saini et al., 85% (35), Ahire et al., 96% (36), and Manizuzzaman et al., 93.4% (37) and 97.53% (15)]. Of note, different machine learning models and EEG features were employed in these studies that may lead to the differences of classification performance. For instance, naïve Bayes, k-nearest neighbor (KNN), and logistic regression models were adopted by Saini (35), while AdaBoost, KNN, naïve Bayes, and RF were employed by Ahire (36), and support vector machine, KNN, multilayer perceptron (MLP), and logistic regression were used by Manizuzzaman (37). These studies collectively demonstrate that machine learning methods can utilize EEG data for identifying ADHD. In fact, a convergent research direction of machine learning analysis of EEG features for ADHD diagnosis is emerging. The superior classification accuracy achieved in this study indicates that machine learning methods using multidimensional EEG features and feature selection algorithms enhance the identification of ADHD.

### 4.2 Altered brain EEG power in ADHD

Children with ADHD have been shown to exhibit increased slow wave power, primarily theta waves, in the frontal and top areas compared to normal control children (38). Increased slow wave power, particularly theta waves, may indicate immature cortical function and delayed inhibitory function, which are suggestive of low arousal within the nervous system. This may be supported further by the increase of theta waves, which might mirror failures in information processing and cognitive control in these regions. Executive functions, including attention control, planning, and decision-making, are primarily associated with the frontal lobe, whereas perception and spatial processing are linked to the top region of the brain. Impairments in these regions may contribute to behavioral symptoms such as attention deficit, excessive activity, and impulsive behavior in child ADHD (39). Abnormal brain electrical activity in specific frequency bands may be the result of neurochemical abnormalities from an EEG perspective (40). For instance, the elevated theta wave power observed in children with ADHD has been postulated to result from decreased dopamine levels in the frontal lobe, leading to functional deficits. This increased theta wave power is considered to reflect inefficiency within the brain to perform tasks. Moreover, a decrease in power within the beta wave band is commonly observed in ADHD children, indicating a reduction in high-frequency brain electrical activity, which may be associated with impairments in higher-order cognitive processing and alertness. These findings are crucial for ADHD diagnosis, as EEG detection offers an objective means to identify abnormal brain activity within specific frequency band, thereby enhancing diagnostic accuracy. Furthermore, understanding the neurochemical mechanisms underlying disorders such as ADHD facilitates the development of targeted therapies aimed at addressing specific deficiencies, like low levels of dopamine. Behavioral and cognitive interventions can serve as complementary approaches to e pharmacological treatments, thus fostering a holistic enhancement of executive functions and attentional control in children with ADHD.

### 4.3 Enhanced TBR in ADHD

The TBR is a cardinal indicator in EEG research into ADHD in EEG readings (31). An elevated TBR reflects an increase in slow theta waves and a decrease of fast beta waves within the brain, potentially indicating high cortical arousal but low cortical operational states (41). This shift in ratio is believed to be the neurophysiological basis for the attention deficits and impulsive behavior seen in people with ADHD. Those could be attributed to a number of mechanisms, which involve several physiological and neurological factors (42–44). This raises in TBR, in cases of ADHD, points to two serious issues. First, it refers to a problem with the prefrontal cortex (45). It is an area of the brain associated with executive functions such as attention control and impulse inhibition. A higher than usual activity of theta waves in this area might mean inefficiency in processing tasks. Second, TBR in ADHD is increased due to abnormalities in the central neurotransmitters, such as dopamine and norepinephrine, which play a crucial role in the top-down control of attention and behavior (45). This pattern of TBR is frequently observed in individuals with ADHD and is of significant clinical importance for diagnosis (46). However, it is important to note that TBR has certain controversies and limitations as a diagnostic tool. While it provides useful insights into ADHD-related brain activity, TBR may be influenced by factors such as age, gender, and individual differences, which can affect its reliability (43). Additionally, specialized equipment and technical expertise are required for accurate TBR measurement, limiting its widespread applicability in routine clinical practice (47). Lastly, the efficacy of this TBR in diagnosing ADHD has been varied in various studies (48). Hence, it cannot stand as a single diagnostic tool. This is frequently combined with other diagnostic measures to enhance the accuracy and comprehensiveness of ADHD diagnoses in clinical settings. As we delve deeper into studying ADHD EEG signals and refine our detection methods, we will pave the way for more reliable and effective approaches to diagnosing and treating ADHD in the future.

### 4.4 Reorganization of whole-brain functional connectivity in ADHD

Several studies have demonstrated marked abnormalities in whole-brain functional connectivity among children with ADHD (49–51). The present study utilized the SHAP algorithm to identify the most critical EEG features, revealing functional connectivity as a distinguishing characteristic. Functional connectivity abnormalities manifest as weakened or strengthened connections in particular circuits between brain regions, impacting attention, impulse control and multitasking abilities (50). In this line, this study is set in a more general framework of dynamic reconfiguration of brain networks in ADHD, who has viewed changes in functional connectivity patterns that have included changes in the strength of connections, reconfiguration of pathways of connectivity, and also adjustments in interaction patterns between functional networks. The MI analysis utilized in this study was crucial for evaluating functional connectivity, as it provided a non-parametric measure of shared information between EEG signals. MI highlighted notable differences in connectivity patterns between children with ADHD and HC, especially in theta and beta rhythms (29). These findings underscore the importance of functional connectivity in characterizing ADHD-related brain dysfunction. A different degree of functional connectivity abnormality in children with ADHD was witnessed (46, 52, 53), which may be responsible for an unbalanced allocation of attentional resources and hence poor task performance (54). Functional connectivity abnormalities are found in ADHD for both long-range and short-range connections with fMRI (55). A weakening of long-range connections can lead to a reduction in information integration across the brain; at the same time, enhanced short-range connections might compensate for these long-range deficits (55). The dynamic reconfiguration of brain networks in children with ADHD reveals complex changes of these patterns. It involves changes in the strength of connections, their pathway reconfiguration, and changes in interaction patterns between functional networks (28). These findings have critical clinical implications, offering insights into the neural mechanisms underlying ADHD symptoms and guiding the development of targeted interventions. Further research is warranted to elucidate the dynamic reconfiguration of brain networks in ADHD and its longitudinal evolution.

### 4.5 Methodological considerations

There are several factors that need to be considered when interpreting the findings of this work. First, it is important to note that due to the limitations of the publicly available dataset used in this study, we are unable to provide information on the demographic and clinical characteristics of the participants (including current treatments, duration of ADHD, cognitive functioning, etc.). This information may not only contribute to a deeper understanding of the neural mechanisms of ADHD and the effectiveness of different treatments, but also may further improve the robustness of the machine learning analysis framework. Therefore, we suggest that future studies should prioritize obtaining these comprehensive participant characteristics when collecting primary data to enrich the study and promote a more nuanced understanding of ADHD. Second, a previously-validated feature selection and cross-validation approach was adopted in this work to mitigate the influence of overfitting. Moreover, the CatBoost model was determined for presenting the main findings. The CatBoost model is capable of reducing the overfitting influence through Symmetric Trees and Ordered Boosting. Nevertheless, the robustness and the generalizability of the results could be benefit from replication in larger independent cohorts.

## 5 Conclusion

In this study, we constructed a machine learning analysis framework through incorporating multidimensional feature extraction and selection framework for ADHD diagnosis and more importantly investigate the underlying neural mechanisms in children with ADHD. The results indicate that the optimal feature subset contains 206 features, corresponding to a superior classification accuracy of 99.58%. Within the optimal feature subset, ADHD exhibit increased power in theta, alpha, and beta rhythms, elevated TBR values, and reorganization of whole-brain functional connectivity across all frequency bands, primarily characterized by enhanced functional connectivity. This study utilizes machine learning to reveal the neural mechanisms of ADHD from a data-driven perspective and provides an analytical framework for ADHD diagnostic research. With further validation on larger external cohorts, the results may lead to practical automatic diagnosis of ADHD based upon objective neuroimaging data.

## Data Availability

Publicly available datasets were analyzed in this study. This data can be found here: https://ieee-dataport.org/open-access/eeg-data-adhd-control-children.
